# Genome sequences of polar *Carnobacterium maltaromaticum* strains 2857 and 2862 with genes for glycerol and 1,2-propanediol pathways

**DOI:** 10.1128/mra.00283-25

**Published:** 2025-05-27

**Authors:** Tamara Aleksandrzak-Piekarczyk, Jakub Grzesiak, Jan Gawor, Katarzyna Kosiorek

**Affiliations:** 1 Institute of Biochemistry and Biophysics, Polish Academy of Sciences, Warsaw, Poland; Montana State University, Bozeman, Montana, USA

**Keywords:** *Carnobacterium maltaromaticum*, psychrotolerant bacteria, 1,2-propanediol biosynthesis, glycerol metabolism

## Abstract

We report genome sequences of two polar *Carnobacterium maltaromaticum* strains: 2857 (draft, 3.54 Mb, 34.4% GC) and 2862 (complete, 3.61 Mb, 34.6% GC, five plasmids). Sequencing used Illumina (both) and Nanopore (2862). Genome analysis revealed genes for glycerol conversion to 1,2-propanediol, suggesting potential for sustainable bioprocessing in cold environments.

## ANNOUNCEMENT

*Carnobacterium* spp. are typically mesophilic but have been isolated from cold and extreme environments (1–3). Here, we report the genome sequences of two psychrotolerant *Carnobacterium maltaromaticum* strains, 2857 and 2862, from Arctic postglacial soils in the foreland of Hans Glacier, Spitsbergen (77.015244 N, 15.597403 E) and from freshwater microbial mats in the Jasnorzewski Gardens, King George Island, Antarctica (62.15943 S, 58.4683 W), respectively (1). These genomes provide insights into *Carnobacterium*’s metabolic potential in extreme cold.

Bacteria were cultured in glucose-M17 broth (Oxoid) (16 hours; 16°C) under aerobic static conditions. Cultures (5  mL in 15  mL tubes) were partially aerated (~30–40% headspace). Genomic DNA from both strains was extracted from 5 mL of culture using a modified cetyltrimethylammonium bromide (CTAB)/lysozyme protocol (4), without the use of commercial kits. Briefly, cells were treated with lysozyme (20  mg/mL, 37°C, 30  min), followed by proteinase K digestion and CTAB-based lysis. DNA was extracted (phenol:chloroform:isoamyl alcohol), precipitated, ethanol washed, and RNase A-treated. The protocol was optimized to preserve high-molecular-weight DNA suitable for long-read sequencing. DNA quality was assessed by spectrofluorometry and gel electrophoresis. Illumina libraries were prepared with NEB Ultra II FS and sequenced on a MiSeq (600-cycle v3, paired-end). Sequence quality was assessed with FASTQC v.0.12.0 (5), and reads trimmed using fastp v.0.23.2 (6). Illumina sequencing yielded 3,537,284 reads (1,032,782 nt) for strain 2857 and 1,919,722 reads (480,405,458 nt) for 2862. The N50 value for the draft assembly of strain 2857 was 211,340  bp. Detailed sequencing and assembly statistics are presented in Table 1.

**TABLE 1:** Sequencing assembly metrics

Sample ID	Genome status	GenBank Acc. nos.	Contigs	Length (bp)	%GC	Sequencing coverage
2862 (chromosome)	Complete	CP185245	1	3,605,406	34.64	130x (Illumina + ONT)
p2862_p1 (plasmid)	Complete	CP185246	1	10,598	33.43	5,204x (Illumina + ONT)
p2862_p2 (plasmid)	Complete	CP185247	1	43,403	35.47	274x (Illumina + ONT)
p2862_p3 (plasmid)	Complete	CP185248	1	60,935	33.23	310x (Illumina + ONT)
p2862_p4 (plasmid)	Complete	CP185249	1	66,284	33.41	145x (Illumina + ONT)
p2862_p5 (plasmid)	Complete	CP185250	1	79,584	32.4	99x (Illumina + ONT)
2857	Draft	JBMVSO010000000	60	3,544,513	34.4	231x (Illumina)

Strain 2862 underwent long-read sequencing on a GridION with an R9.4.1 flow cell. Genomic DNA was sheared (~30 kb, 26G needle) and size-selected using the Short Read Eliminator kit (Circulomics) to enrich for fragments > 10 kb. Size selection was performed via precipitation-based removal of short DNA fragments, following the manufacturer’s protocol. Libraries were prepared with SQK-LSK109 and EXP-NBD103 kits (Oxford Nanopore Technologies). Basecalling (Guppy v.6.1.3, super accuracy) yielded 14,922 reads (212,795,885 nt, N50 = 18 kb). Data were filtered (NanoFilt v.2.8.0, QV <12, reads <1 kb removed) (7), adapters trimmed (Porechop v.0.2.4), and quality-checked with NanoPlot v.1.41.6. Trycycler v.0.5.3 (8) was used for assembly, integrating consensus assemblies generated by Flye v.2.9, Unicycler v.0.4.8, Raven v.1.8.1, and Miniasm v.0.3-r179. As part of this pipeline, Trycycler automatically identified and trimmed terminal overlaps, generating circular consensus sequences. The genome was polished with Medaka v.1.7.2, Polypolish v.0.5.0 (9), and PyPolca v.0.3.1 (10, 11) (). Validation was done with GAEP v.1.2.3, and genomes were rotated at *dnaA* or *repA* using dnaapler v.1.2.0. Annotation was performed using PGAP v.6.6 (12). Genome completeness was confirmed by the successful circularization of all replicons and validation with GAEP, which indicated no gaps or missing core genes. Default parameters were used for all bioinformatic software unless otherwise noted.

Both strains metabolized glycerol, a feature observed in some *Carnobacterium* spp. (1). Genomic analysis revealed the presence of genes for glycerol metabolism, including those encoding glycerol dehydrogenase (EC 1.1.1.6), glycerol kinase (EC 2.7.1.29), and glycerol-3-phosphatase (EC 2.7.1.121). In addition, genes associated with the methylglyoxal pathway were identified, potentially enabling the conversion of glycerone-P to 1,2-propanediol.

## Data Availability

The complete genome sequence of *C. maltaromaticum* 2862, including its chromosome and plasmids (p2862_p1, p2862_p2, p2862_p3, p2862_p4, p2862_p5), and the draft genome sequence of *C. maltaromaticum* 2857 have been deposited in GenBank under accession numbers CP185245, CP185246, CP185247, CP185248, CP185249, CP185250, and JBMVSO010000000. Raw sequencing reads are available in the NCBI Sequence Read Archive (SRA) under the following accession numbers: *C. maltaromaticum* 2862 Illumina reads (SRR32776913), Oxford Nanopore reads (SRR32776912), and *C. maltaromaticum* 2857 Illumina reads (SRR32769006). The corresponding BioProject and BioSample accession numbers are PRJNA1237908/SAMN47442554 (*C. maltaromaticum* 2862) and PRJNA12378782/SAMN47444522 (*C. maltaromaticum* 2857).
